# T-cell states in aortic dissection: from molecular immune profiling toward causal and translational validation

**DOI:** 10.1080/07853890.2026.2753327

**Published:** 2026-10-29

**Authors:** Xiang Ye, Yihao Wang, Yeting Li, Hao Zhu, Xia Li, Jianxiang Song

**Affiliations:** a Northern Jiangsu Institute of Cardiovascular Disease Prevention and Treatment, Jiangsu University, Yancheng, PR China; b Department of Thoracic Surgery, Yancheng Third People’s Hospital, Affiliated Hospital 6 of Nantong University, Yancheng, PR China; c Medical School, Nantong University, Nantong, PR China; d Department of General Medicine, Affiliated Hospital 6 of Nantong University, Yancheng Third People’s Hospital, Yancheng, PR China

**Keywords:** Aortic dissection, T lymphocytes, single-cell RNA sequencing, vascular inflammation, translational medicine

## Abstract

**Background/Objectives:**

Single-cell RNA sequencing and T-cell receptor profiling reveal substantial T-cell heterogeneity in aortic dissection (AD), but whether these responses contribute to vascular injury or reflect post-onset immune activation remains uncertain. This review evaluates human and experimental evidence by disease stage, tissue compartment, functional validation, T-cell specificity, and intervention timing, distinguishing descriptive profiling from evidence supporting causal and translational inference.

**Discussion:**

Human studies identify Th1/Th17-associated, CD8^+^ cytotoxicity-associated, and regulatory T-cell alterations, with stage-related differences in T-cell composition and clonality. Most evidence is cross-sectional and obtained after clinical onset. Antigen specificity, direct aortic-wall cytotoxicity, impaired Treg suppression, and T-cell-mediated aortic-wall injury remain unproven. Single-cell and TCR profiling identify candidate T-cell states and clonotypes for spatial and functional validation, not causal proof. Among candidate pathways, the IL-6–STAT3–IL-17 axis currently has the strongest AD-specific preclinical rationale, but existing interventions are largely non-T-cell-restricted and initiated before or during disease induction. Stronger causal attribution requires convergent human evidence, post-lesion T-cell-restricted perturbation, disease-progression endpoints, and rescue or target-engagement studies. The subacute phase after anatomical and haemodynamic stabilisation may offer a window to study persistent injury-amplifying T-cell responses, whereas early acute and perioperative immunomodulation remains unsupported.

**Conclusions:**

Current evidence supports altered T-cell states after AD onset but does not establish an initiating role. Near-term priorities should be prospective biomarker validation and post-lesion mechanistic studies rather than T-cell-directed immuno­modulation during the early acute and perioperative phases. Clinical translation should await reproducible associations linked to stage-specific causal mechanisms and acceptable safety.

## Graphical Abstract

**Figure:**
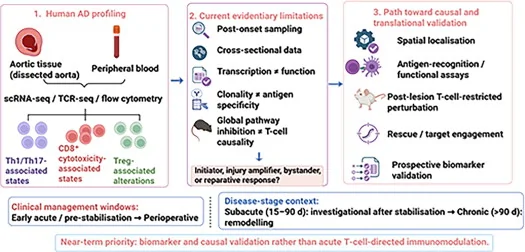

## Introduction

Aortic dissection is a life-threatening condition characterised by separation within the aortic media, usually following an intimal tear that permits pressurised blood to enter the aortic wall and create a false lumen [1–4]. Traditional studies focused primarily on medial structural degeneration and haemodynamic stress [5,6]. More recent work has implicated inflammatory signalling, vascular-wall injury and remodelling in AD [7–11]. Single-cell studies of ascending thoracic aortic aneurysm provide relevant disease context [12], whereas studies of human AD have begun to characterise immune-cell heterogeneity within dissected aortic tissue [13,14]. A small stage-stratified, cross-sectional single-cell study reported differences in T-cell composition, transcriptional programmes and TCR clonotype distribution across acute, subacute and chronic AD samples [15]. For review-level comparisons, hyperacute and acute cases were grouped as ≤14 days from symptom onset, whereas subacute and chronic AD were defined as 15–90 days and >90 days, respectively. These findings do not demonstrate longitudinal phenotypic changes within individual patients. Accordingly, these observations require validation in larger, spatially resolved and longitudinal cohorts. In this review, T-cell composition and transcriptional phenotypes are used as the principal descriptive terms. Cell abundance, transcriptional signatures, inferred effector potential and TCR clonality are treated as distinct evidence domains. Clonal expansion may be assessed using bulk or single-cell TCR-repertoire sequencing. Assignment of expanded clonotypes to specific transcriptional states requires paired single-cell RNA and TCR data. Transcriptional signatures, TCR clonality and cross-sectional differences are valuable for hypothesis generation but are treated as descriptive evidence unless supported by direct functional or longitudinal validation. Accordingly, single-cell profiling is used here to identify candidate T-cell states and clonotypes for subsequent spatial and functional validation rather than as evidence of causality. AD studies have identified metabolic abnormalities at the tissue and circulating T-cell levels, but direct evidence for T-cell-specific metabolic flux or chromatin regulation within the aortic wall remains lacking [16–20]. Mechanistic interpretation therefore still relies substantially on non-AD inflammatory settings, and these metabolic and epigenetic pathways should be regarded as testable hypotheses rather than established T-cell mechanisms in AD [21–27]. The extent to which these post-onset findings vary according to disease stage, reflect reactive immune recruitment, or contribute to subsequent vascular remodelling remains unresolved [13–15]. Available studies suggest enrichment or activation of pro-inflammatory T-cell programmes, including Th1, Th17, and cytotoxic CD8^+^ T-cell-associated signatures, together with altered regulatory T-cell features [13–15,28,29]. However, the magnitude and direction of these changes may vary according to disease stage, tissue compartment, and analytical strategy. Adaptive-innate immune crosstalk may further sustain cytokine- and protease-driven inflammation [30–35]. In severe AD, local vascular inflammation may coexist with systemic inflammatory activation, which is also influenced by tissue ischaemia, malperfusion, surgical trauma, and other clinical stressors [36–39]. This review examines microenvironmental factors associated with differences in T-cell composition, the potential roles of major T-cell subsets, adaptive-innate immune interactions, and the translational implications of these findings. This review focuses primarily on thoracic aortic dissection, particularly acute type A disease where evidence is available. Findings from abdominal aortic aneurysm, atherosclerosis, hypertension, and non-vascular inflammatory diseases are discussed as mechanistic analogies rather than direct evidence for AD [40–45]. This structured narrative review uses an evidence-category framework to distinguish observations in human AD from experimental AD findings and mechanistic extrapolations from related vascular or inflammatory diseases. The central question is therefore not simply which T-cell states are present in AD, but whether any of these states carry causal or clinically informative signals beyond established tissue injury. We then assess whether specific T-cell compositional changes and transcriptional phenotypes satisfy key criteria for causal involvement, with particular attention to disease stage, tissue compartment, temporal precedence, and the possibility that immune activation follows rather than precedes medial injury. Recent reviews have primarily summarised T-cell subsets and candidate adaptive immune mechanisms in thoracic aortic aneurysm and dissection [46]. Rather than providing another subset-based overview, the present review focuses on the distinction between association and causality. We evaluate T-cell findings according to disease stage, tissue compartment, temporal relationship to lesion formation, T-cell specificity, and timing of experimental intervention. This framework is intended to define which observations remain descriptive, which support mechanistic inference, and which require further validation before translational consideration. The novelty of this review lies in defining the evidentiary transition from molecular association to causal and translational validation, rather than providing another catalogue of T-cell subsets or pathways. Neither an initiating nor a post-onset injury-amplifying T-cell function has been established in AD (Figure 1).

**Figure 1.**
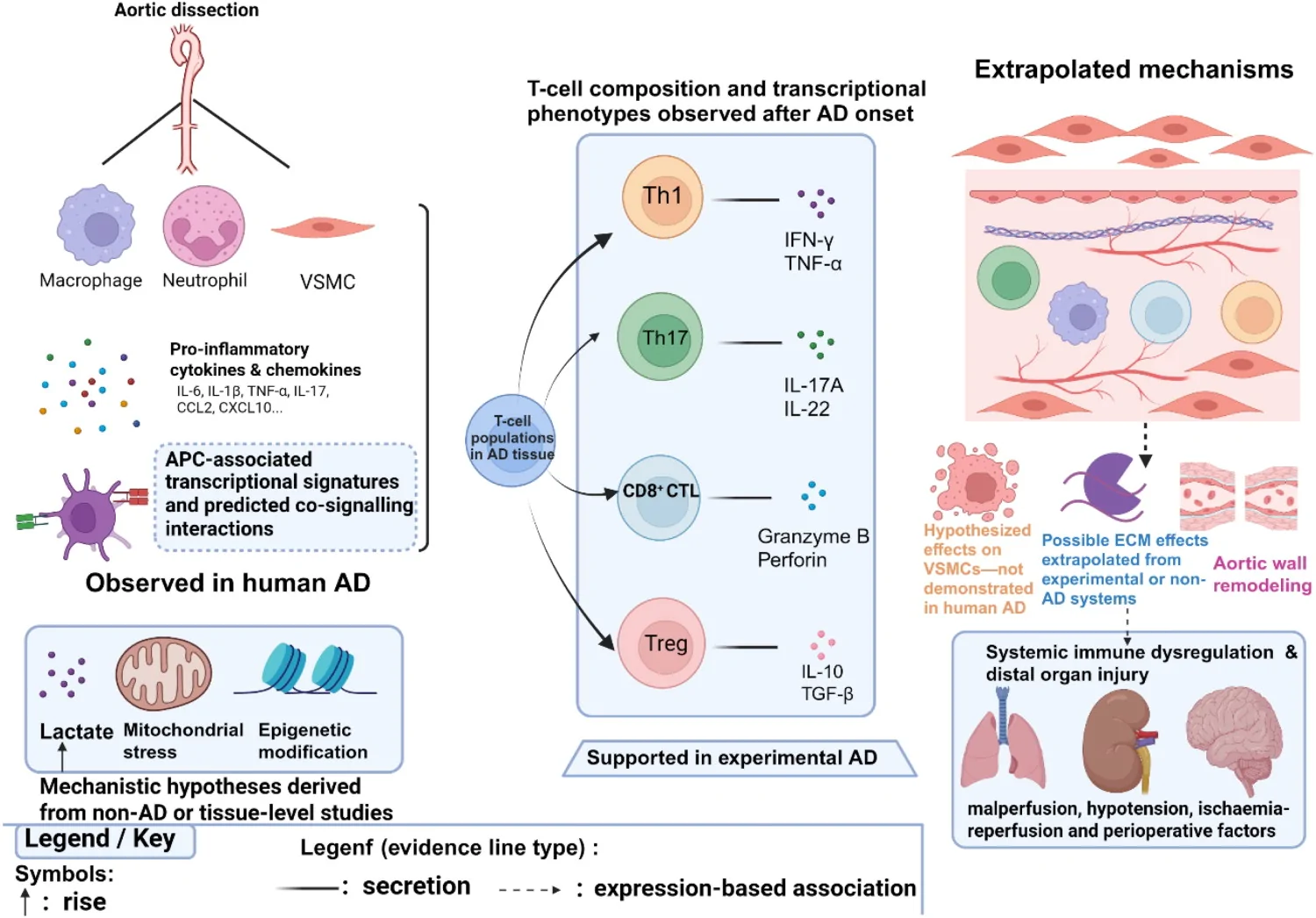
Evidence-category overview of T-cell composition and transcriptional phenotypes associated with aortic dissection.

## Methods: literature search and evidence framework

This structured narrative review searched PubMed and Web of Science Core Collection from database inception to 10 October 2026. Retrieved records were assessed for relevance to the review question based on their titles, abstracts, and available study information, with particular attention to study population, disease context, and T-cell-related findings. Human AD studies were eligible for inclusion when they examined T-cell phenotypes, function, clonality, or clinical associations in aortic tissue or peripheral blood. Experimental studies were included when they investigated T-cell-associated pathways or mechanisms in disease-relevant AD models. Studies without relevant T-cell-related findings or without direct relevance to AD were excluded from the AD-specific evidence synthesis. Studies from non-AD settings were retained only when they provided essential mechanistic context and were not classified as direct AD evidence. For each included study, disease stage, tissue compartment, analytical method, T-cell population, and, where applicable, intervention specificity and timing were considered during evidence appraisal. Evidence categories were assigned at the level of individual findings rather than entire studies. Full database-specific search strategies are provided in Supplementary Table S1.

Disease stage was classified as hyperacute (<24 h), acute (1–14 days), subacute (15–90 days), or chronic (>90 days) [4]; hyperacute and acute cases were grouped as ≤14 days for review-level comparisons. Human findings were assigned to complementary, non-hierarchical evidence categories: H1, direct functional assessment of patient-derived T cells; H2, histological, flow-cytometric, or spatial analysis of human AD tissue; H3, transcriptomic, TCR-repertoire, or computational analysis of human AD tissue; and H4, descriptive or associative peripheral-blood findings after clinical onset. Classification was performed at the finding level, allowing one study to contribute to multiple categories. H1 was retained as the benchmark for direct human functional evidence but was not considered mandatory for translational progression. Experimental evidence was additionally assessed according to T-cell specificity and intervention timing. Where direct aortic-wall functional evidence was unavailable, causal confidence was judged from convergent human findings, disease-relevant models, post-lesion T-cell-restricted perturbation, and rescue or target-engagement evidence. Evidence was synthesised qualitatively without meta-analysis or formal risk-of-bias assessment. Study-specific temporal definitions were retained when they differed from this framework or when exact symptom-onset intervals were unavailable.

### Microenvironmental correlates and candidate determinants of T-cell composition and transcriptional phenotypes in aortic dissection

#### Imbalance of local cytokine and chemokine networks

Human AD studies have separately reported IL-6-associated systemic or aortic-wall inflammation and altered circulating Th17 frequencies or IL-17A-associated signals. These findings arise from different cohorts and tissue compartments and do not establish an IL-6-STAT3-Th17 axis within AD-infiltrating T cells [28,29,47–50]. In Ang II-induced murine models, IL-6 deficiency, IL-17A neutralisation or deficiency, and pharmacological STAT3 inhibition reduced dissection-related phenotypes and inflammatory-cell recruitment [51]. Separately, an adventitial IL-6/MCP1 amplification loop was implicated in macrophage-mediated vascular inflammation and aortic dissection [52]. The interventions evaluated by Ju et al. [51] were not T-cell-restricted and were initiated during disease induction, limiting inference regarding a post-lesion T-cell-specific effect. They therefore provide pathway-level evidence but do not demonstrate a post-onset T-cell-mediated injury-amplifying function. In the BAPN-plus-Ang II model, global Il17a deficiency did not materially alter the baseline AD phenotype under normal-salt conditions but prevented the additional worsening induced by high-salt exposure. Because Il17a deficiency also altered aortic ECM properties before disease induction, this study does not isolate a post-onset T-cell effector mechanism [53]. General immunology studies indicate that metabolic programmes influence effector and regulatory T-cell fate and function [54,55]. However, these studies do not establish an IL-6-STAT3-dependent metabolic mechanism in AD-infiltrating T cells. Direct IL-17A-induced matrix degradation or VSMC phenotypic alteration has likewise not been demonstrated in human AD-derived vascular cells. The evidence supporting the proposed IL-6–STAT3–IL-17 axis, together with its major limitations, is summarised in Figure 2.

**Figure 2.**
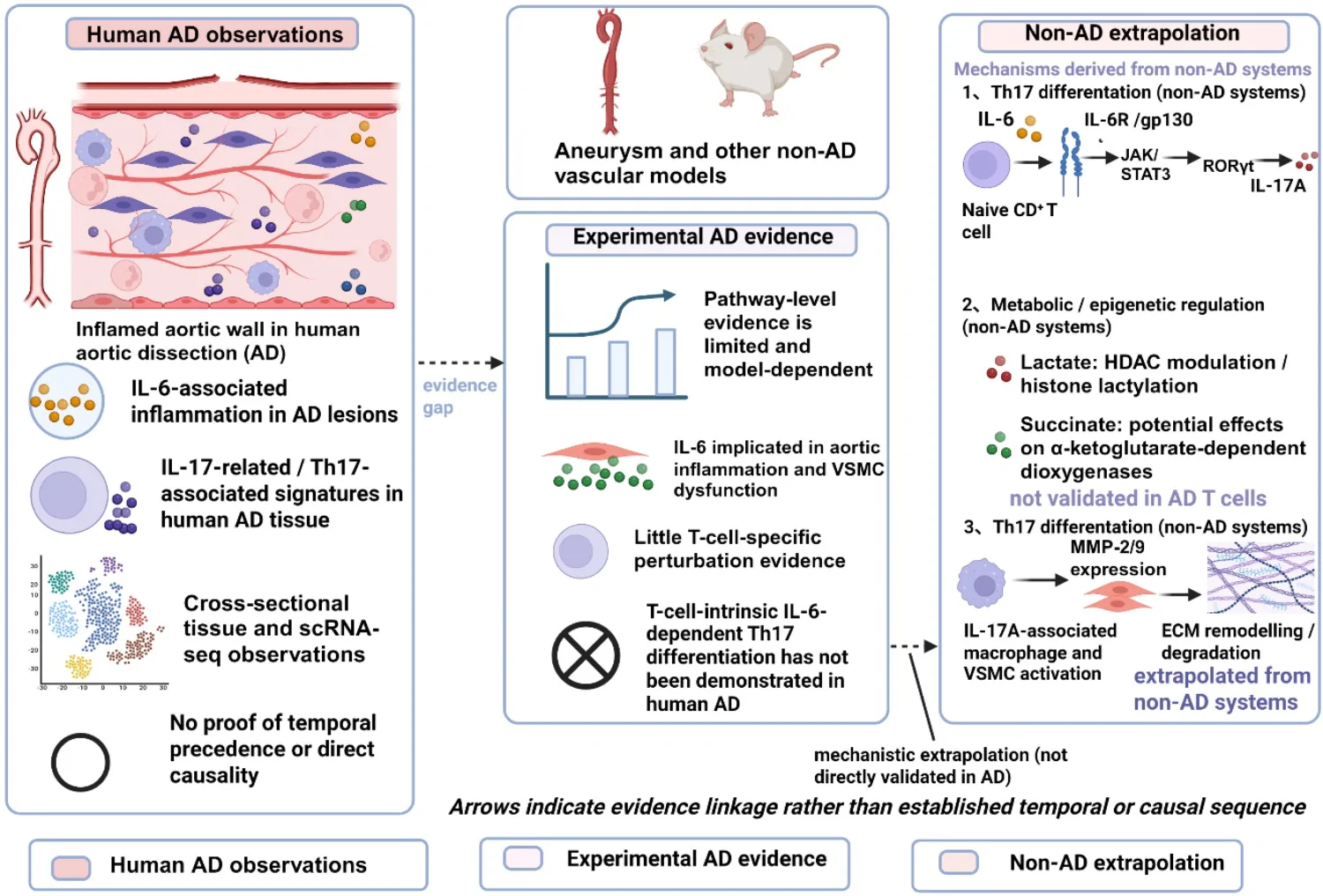
Evidence map of the proposed IL-6–STAT3–IL-17 axis in aortic dissection.

In non-AD vascular models, IFN-γ has been associated with reduced expression of VSMC contractile markers and altered matrix-related programmes. IFN-γ derived from Th1 and CD8^+^ T cells may also enhance pro-inflammatory macrophage activity, whereas macrophage-derived IL-12 and IL-18 can support IFN-γ production in other inflammatory settings. Whether this potential feed-forward interaction occurs in AD or contributes to matrix injury remains unknown [56–58].

Chemokine expression may shape leukocyte recruitment to AD lesions. CCL2 is best supported as a monocyte/macrophage-recruiting signal, whereas evidence for direct recruitment of naive T cells in AD is limited. CCL2 expression has been reported in inflamed aortic tissue and may promote CCR2-dependent monocyte recruitment [52]. Recruited macrophages may subsequently contribute to local IL-6 and TNF-α production, although the extent to which this pathway regulates T-cell recruitment, subset composition or transcriptional phenotypes in human AD remains unclear. CXCL1 may contribute indirectly to a Th17-favouring environment through neutrophil recruitment and cytokine release; direct evidence in AD remains limited. CXCL10-CXCR3 and CXCL16-CXCR6 have been proposed as candidate pathways for T-cell recruitment, but direct functional validation in human or experimental AD is lacking [44,46]. Collectively, dysregulated cytokine and chemokine signalling may influence T-cell recruitment and differentiation through pathways such as JAK-STAT and NF-κB. However, the relative contribution of individual mediators has not been established in human AD [46,56]. The candidate chemokine–leukocyte recruitment pathways and the relative strength of evidence supporting each pathway are summarised in Figure 3.

**Figure 3.**
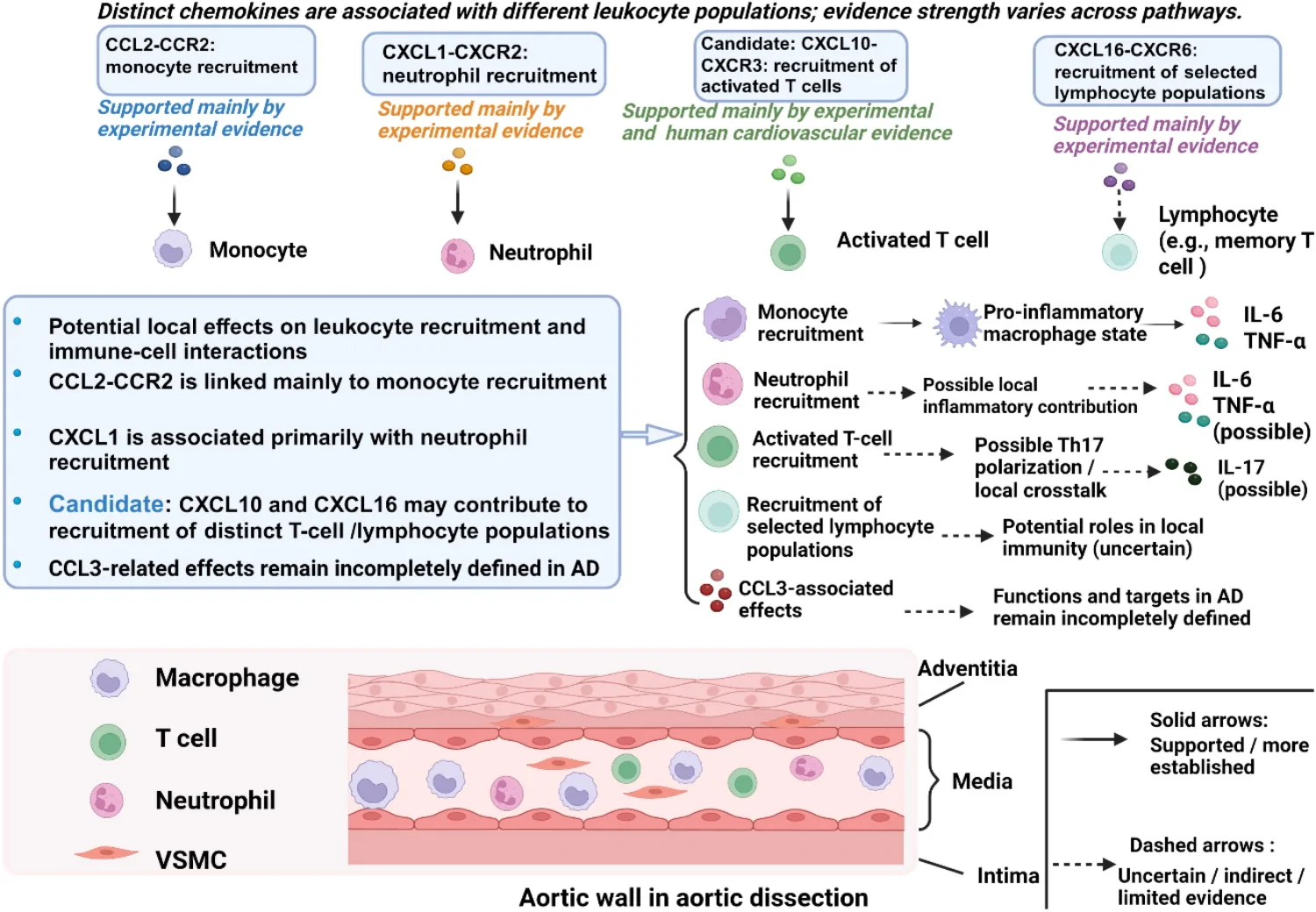
Candidate chemokine-associated leukocyte recruitment pathways in the aortic dissection microenvironment. Direct functional validation of the proposed T-cell recruitment pathways remains lacking.

Recent scRNA-seq studies of human AD tissue have reported stage-associated heterogeneity in T-cell composition and activation-associated transcriptional phenotypes. However, stage-specific analyses were based on small patient subgroups and require validation in larger cohorts. In a cross-sectional, stage-stratified dataset, a transcriptionally annotated CD8^+^ effector-like T-cell population showed elevated cytotoxicity-associated gene-set scores, particularly in subacute samples. These findings do not demonstrate longitudinal expansion within individual patients. CD4^+^ effector-memory and central-memory T-cell populations and several computationally inferred immune-vascular ligand-receptor interactions were also reported.

#### Potential involvement of antigen presentation and co-signalling

AD tissue contains antigen-presenting cells and T-cell populations with activation-associated transcriptional signatures, and inflammatory conditions may alter local co-stimulatory and co-inhibitory signalling. Mechanical injury and matrix degradation may release endogenous molecules that could serve as candidate antigens. However, recognition of these molecules by AD-associated T cells has not been demonstrated. No defined aortic autoantigen, antigen-specific TCR response, or functionally validated autoreactive T-cell response has been demonstrated. Expanded clonotypes identified by bulk or single-cell TCR-repertoire sequencing indicate nonuniform repertoire expansion or selective enrichment. They do not identify the relevant antigen, demonstrate that expansion occurred within the aortic wall or establish aortic autoreactivity. Expanded clones may have arisen in response to unrelated prior antigens and subsequently entered the lesion [59–61]. Whether CD80/CD86-CD28 or PD-1/PD-L1 signalling is functionally dysregulated in AD lesions remains unknown. Paired scRNA-seq and TCR sequencing can serve as a discovery step to identify candidate T-cell states and expanded clonotypes for further investigation. Causal or antigen-specific attribution, however, requires subsequent spatial localisation, candidate-antigen stimulation, MHC-restricted recognition assays, and functional target-cell validation [62–64]. Antigen-driven expansion and cytokine-driven bystander activation should therefore be treated as competing explanations rather than sequential steps in an assumed autoimmune mechanism. Even spatial enrichment of an expanded clonotype within the aortic wall would establish localisation, not antigen identity. Evidence for antigen-driven vascular injury would require concordant TCR expansion, lesion localisation, MHC-restricted recognition and a measurable target-cell response. A stepwise experimental framework for establishing antigen specificity, MHC-restricted recognition, and target-cell effects in AD is outlined in Figure 4.

**Figure 4.**
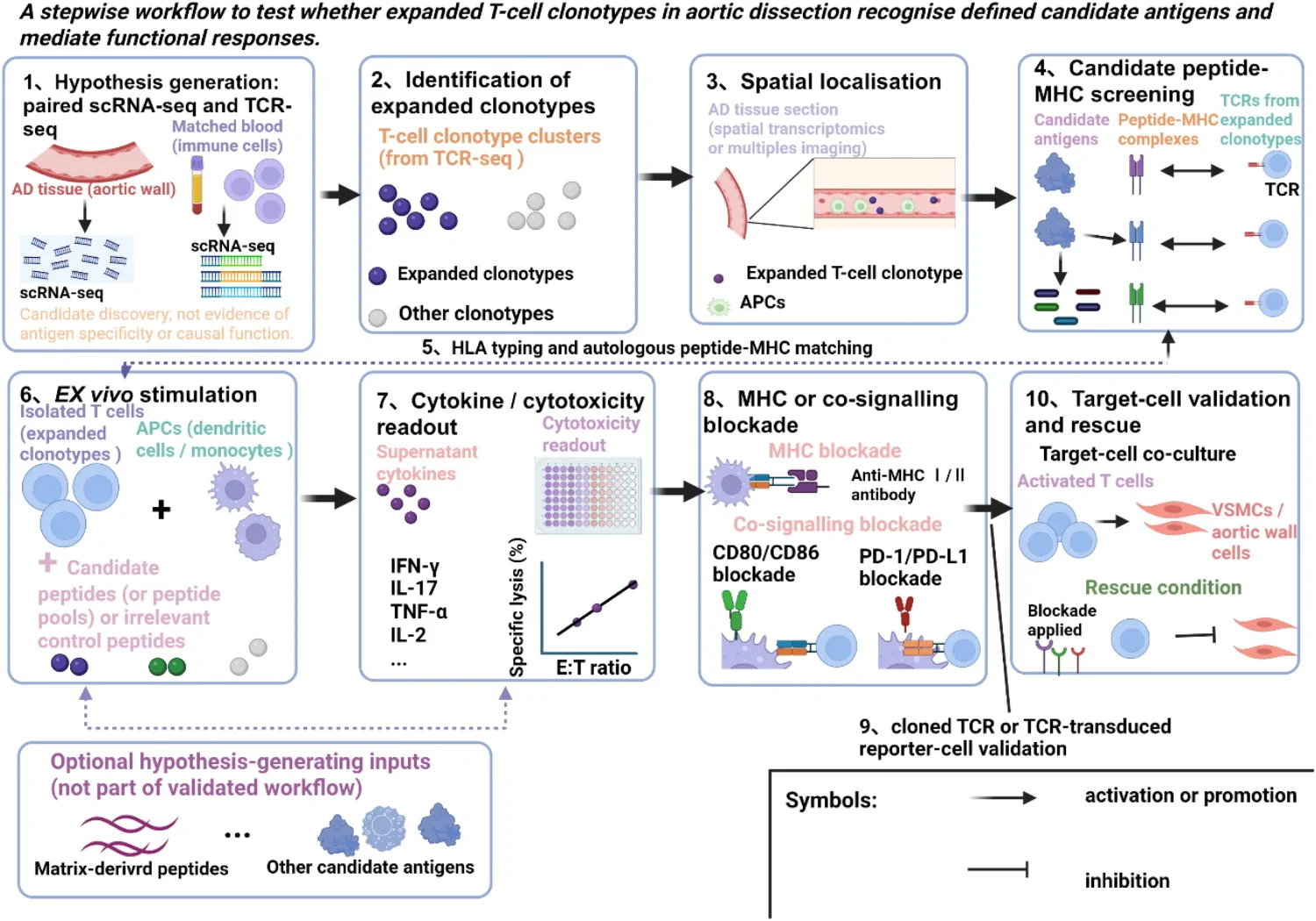
Hypothesis-generation and causal-validation framework for testing antigen specificity and target-cell effects of T-cell responses in aortic dissection.

#### Metabolic and epigenetic hypotheses for T-cell state regulation

One peripheral-blood study reported CD36-associated lipid uptake, ferroptosis-related changes and impaired activation of CD4^+^ T cells in Acute Type A Aortic Dissection [16]. By contrast, other AD studies have mainly examined mitochondrial, glucose or energy-metabolic abnormalities at the tissue or VSMC level [17–20]. Based largely on findings from cancer, autoimmunity, and other inflammatory disorders, metabolic flux, spatial metabolite availability and chromatin regulation in aortic-wall T cells remain uncharacterized [21–27]. Existing studies identify tissue-level metabolic signatures, but not T-cell-specific metabolite concentrations, metabolic flux or chromatin changes. These mechanisms should therefore be framed as experimental priorities rather than components of an established disease model.

### T-cell subset-associated findings and proposed effects on aortic wall cells

Human AD studies report Th17-associated transcriptional signatures, CD8^+^ T-cell populations with cytotoxicity-associated gene expression, and alterations in Treg abundance or regulatory transcriptional signatures. These observations do not establish cytokine secretion, degranulation, suppressive capacity or direct vascular-cell injury. Proposed effects on VSMCs and ECM homeostasis are derived mainly from experimental AD and non-AD vascular models; direct functional evidence in human AD remains limited [46,47,53,65,66]. Human peripheral-blood studies have reported altered helper-T-cell distributions, lymphopenia and impaired CD4^+^ T-cell activation or proliferative capacity after AD onset [16,28,29]. These studies provide direct evidence of systemic T-cell perturbation but do not localise the abnormality to the dissected aortic wall. Lymphocyte redistribution, neuroendocrine stress, malperfusion, tissue ischaemia and sampling before or after surgery remain major competing explanations. The principal human AD studies are summarised in Table 1, while Table 2 presents the major T-cell-associated findings, their highest admissible inference, and key evidence gaps. Detailed experimental evidence from AD models and mechanistic findings from non-AD settings are provided in Supplementary Table S2.

**Table 1.** Principal human studies evaluating T-cell-associated alterations in aortic dissection.

Study	Disease stage (time from symptom onset)	Sample source	Method	Main T-cell-related finding	Evidence category
Han et al. 2024 [13]	Acute (study-defined; exact time from symptom onset not reported)	Aortic tissue	scRNA-seq	Identified immune-cell heterogeneity and T-cell-associated transcriptional alterations in dissected aortic tissue.	H3
Negoto et al. 2024 [14]	Not clearly reported.	Aortic tissue	Immunostaining with quantitative immune-cell analysis.	Reported site-specific infiltration and associations among helper T-cell subsets and other immune-cell populations in human AD tissue.	H2
Zhao et al. 2025 [15]	Acute (<14 d), subacute (15–90 d), and chronic (>90 d), study-defined.	Aortic tissue	scRNA-seq with TCR-repertoire analysis	Demonstrated stage-associated differences in T-cell composition, cytotoxicity-associated transcriptional states and clonotype distribution.	H3
Li et al. 2024 [16]	Acute (study-defined; exact time from symptom onset not reported)	Peripheral blood	Cellular and molecular analysis of CD4⁺ T cells	Reported CD36-associated lipid uptake, ferroptosis-related changes and impaired CD4⁺ T-cell activation.	H4 for descriptive peripheral-blood findings; H1 for direct functional assays of patient-derived CD4⁺ T cells.
Ye et al. 2018 [28]	Predominantly acute; median 24 h (IQR 12–34 h) from chest-pain onset to blood sampling.	Peripheral blood	Circulating T-cell subset analysis.	Reported altered circulating Th1, Th2, Th9, Th17, Th22 and Treg distributions after AD onset.	H4
Song et al. 2022 [29]	Not clearly reported.	Peripheral blood	T-cell subset quantification and clinical association analysis	Th17-related measures were associated with inflammatory markers and mortality risk	H4
Liu et al. 2019 [73]	Acute (study-defined; exact time from symptom onset not reported)	Peripheral blood	Circulating Th17 analysis with longitudinal clinical follow-up	Circulating Th17 levels were associated with subsequent clinical outcomes	H4
Zong et al. 2023 [74]	Not clearly reported.	Peripheral blood	MALT1 and helper-T-cell analysis	MALT1 was associated with Th1/Th17 measures and 30-day mortality	H4

*Note:* H1–H4 are complementary, non-hierarchical evidence categories assigned at the level of individual findings rather than entire studies. H1 denotes direct functional assessment of patient-derived T cells irrespective of tissue source; H2, histological, flow-cytometric, or spatial analysis of human AD aortic tissue; H3, transcriptomic, TCR-repertoire, or computational analysis of human AD tissue; and H4, descriptive or associative peripheral-blood findings after clinical onset. A single study may contribute to multiple categories. H1-level human evidence remains limited. Li et al. ^[^^16^^]^ provided direct functional evidence in patient-derived peripheral-blood CD4^+^ T cells, including activation and proliferation assays. However, no human AD study has directly demonstrated the functional effects of aortic-wall T cells on vascular target cells or established a T-cell-mediated aortic-wall injury mechanism. Disease stages follow the definitions provided in the Methods. When exact symptom-onset intervals were unavailable, the original study’s designation was retained and explicitly indicated in the table.

**Table 2.** Summary of T-cell-associated findings and evidence gaps in aortic dissection.

T-cell finding	Human AD evidence	Highest admissible inference	Key evidence gap
Th17/IL-17-associated phenotype	Th17/IL-17-related signals have been reported in human aortic tissue and peripheral blood after AD onset [14,28,29].	Association with established inflammation is supported; a T-cell-specific causal role remains unproven.	Post-lesion, T-cell-restricted Rorc/Il17a perturbation and direct Th17–aortic-wall functional assays.
CD8⁺ cytotoxicity-associated phenotype	Human AD tissue contains CD8⁺ T-cell populations with cytotoxicity-associated transcriptional programmes [15].	A post-onset cytotoxicity-associated phenotype is supported, but direct vascular-cell killing is unproven.	CD8⁺ T-cell-specific perturbation, antigen recognition and aortic target-cell killing assays.
Th1/IFN-γ-associated phenotype	Th1-associated tissue findings and altered circulating helper-T-cell distributions have been reported [14,28,29].	Mechanistic plausibility only; a T-cell-specific causal role has not been established.	Post-lesion T-cell-restricted IFN-γ perturbation and direct aortic-wall cell assays.
Treg-associated phenotype	Altered Treg abundance or regulatory features have been reported in aortic tissue [14] and peripheral blood [28].	Regulatory imbalance is possible, but suppressive dysfunction and lineage instability remain unproven.	Functional suppressive assays and stage-specific Treg perturbation with rescue.
Antigen-specific immunity	No defined aortic autoantigen or functionally validated antigen-specific T-cell response has been demonstrated.	Antigen-specific autoimmunity remains hypothetical.	Paired scRNA/TCR, spatial clonotype mapping, peptide–MHC testing and target-cell recognition assays.
Metabolic/epigenetic alterations	Peripheral-blood CD4⁺ T-cell metabolic abnormalities have been reported [16], but aortic-wall T-cell metabolic or chromatin data are lacking.	Circulating CD4⁺ T-cell dysfunction is supported; an aortic-wall T-cell metabolic or epigenetic mechanism remains unestablished.	Aortic-wall T-cell metabolomic, flux and chromatin profiling followed by cell-restricted perturbation.

*Note:* H1–H4 are complementary, non-hierarchical evidence categories assigned at the level of individual findings; a single study may therefore contribute to more than one category. Direct functional assessment of patient-derived T cells is classified as H1 irrespective of tissue source. Transcriptional signatures, computational ligand–receptor analyses and TCR clonality are considered descriptive unless supported by functional validation. Detailed category definitions are provided in the Methods and Table 1.

#### Potential effects of Th1 and Th17-associated cytokines on aortic wall cells

IFN-γ- and IL-17A-associated vascular effects are supported mainly by non-AD vascular models or global experimental perturbation [51,53,56–58]. Direct effects of AD-derived T-cell mediators on aortic VSMCs have not been demonstrated. Disease-relevant co-culture or conditioned-medium experiments with cytokine neutralisation are therefore required to establish a T-cell-to-aortic-wall effector mechanism.

#### Evidence for cytotoxicity-associated CD8^+^ T-cell phenotypes

Human AD tissues contain CD8^+^ T-cell populations expressing cytotoxicity-associated transcriptional programmes [13,15]. These findings are consistent with a cytotoxicity-associated transcriptional phenotype but do not demonstrate degranulation, antigen-specific recognition or direct killing of VSMCs or other aortic wall cells. Clonotype expansion should be reported separately and only for cohorts analysed by TCR sequencing. Within the literature reviewed here, no study has established an aortic target antigen, MHC-restricted recognition, autologous VSMC killing or rescue of vascular injury through CD8^+^ T-cell-specific depletion in a disease-relevant AD model. Perforin-granzyme and Fas-FasL-mediated vascular-cell injury mechanisms derive from non-AD models and should not be presented as AD-specific [42,67]. Accordingly, human AD data support the presence of post-onset CD8^+^ T-cell populations with cytotoxicity-associated transcriptional phenotypes, but not validated cytotoxic function. These findings are compatible with several post-onset models, including secondary recruitment, bystander activation, inflammatory persistence, and a possible—but untested—injury-amplifying response. Expression of GZMB, PRF1, NKG7 or other cytotoxicity-associated genes should not be interpreted as evidence that CD8^+^ T cells kill aortic wall cells *in vivo*.

#### Altered Treg abundance and regulatory transcriptional signatures

Altered Treg abundance or regulatory transcriptional signatures have been reported in AD [14,28], but suppressive capacity, FOXP3 stability and lineage fate have not been directly tested. Evidence for Treg plasticity derives mainly from autoimmune and chronic inflammatory disorders [68–72]. Whether comparable lineage instability occurs in AD remains unknown and will require lineage-informed functional and spatial analyses. Direct *in vivo* evidence for this process in human AD is lacking. Such plasticity, if present, could theoretically alter local inflammatory regulation and matrix remodelling. However, neither Treg lineage conversion nor a consequent injury-amplifying effect has been demonstrated in AD. No evidence demonstrates that Treg deficiency or dysfunction precedes dissection onset. Post-onset changes may reflect redistribution, activation-associated loss, compensatory recruitment or systemic stress.

### Pathogenic significance and temporal context of T-cell alterations in aortic dissection

Observed T-cell changes may represent pathogenic, bystander, reparative or resolution-associated responses. Current cross-sectional transcriptomic data cannot distinguish among these possibilities or establish whether T-cell activation precedes medial injury [13–15].

#### Competing temporal models of T-cell involvement in aortic dissection

T-cell accumulation within AD tissue and stage-associated inflammatory transcriptional profiles are compatible with a disease-promoting role but are insufficient to establish it. This question cannot be resolved by interventions that broadly suppress inflammatory pathways, particularly when they are not T-cell specific or are initiated before lesion establishment [51–53]. Current human data are compatible with several non-exclusive temporal models of T-cell involvement in AD. An initiating model would require evidence that a defined T-cell response precedes medial disruption, for which no direct human evidence currently exists. A second possibility is post-lesion amplification: T cells recruited after medial injury may sustain inflammation or promote subsequent wall degeneration without participating in the initial dissection event. This model is biologically plausible but has not been tested using delayed, T-cell-restricted intervention. A third explanation is bystander activation driven by tissue necrosis, intramural haemorrhage, cytokine release, malperfusion or systemic stress. Finally, some T-cell populations may participate in inflammatory resolution or vascular repair rather than injury. Distinguishing these possibilities will require longitudinal assessment and functional studies linked to disease progression.

#### T-cell responses secondary to aortic wall injury

T-cell activation may arise secondary to medial injury, intramural haemorrhage, release of damage-associated signals and systemic stress. Most human aortic samples are obtained during emergency surgery after dissection has already occurred. Tissue acquisition is therefore constrained by post-lesion sampling, specimen fragmentation, spatial heterogeneity, and variable tissue viability, which limit viable immune-cell isolation, paired autologous functional assays, and serial tissue sampling. Tissue necrosis, malperfusion, and other systemic stressors may further alter T-cell recruitment and activation [36–39]. Consequently, the observed T-cell changes may be secondary to established injury. A post-onset injury-amplifying role remains possible but untested and cannot be inferred from tissue enrichment alone. Importantly, recruitment after lesion formation does not necessarily imply biological irrelevance. A secondary T-cell response could still contribute to subsequent wall degeneration or remodelling. The critical distinction is therefore between temporal origin and functional consequence: a response may be post-onset yet still be pathogenic during disease progression.

#### Stage-specific interpretation of T-cell alterations

Using the temporal definitions adopted in this review, the ≤14-day group, comprising hyperacute and acute AD, is particularly susceptible to confounding by tissue necrosis, haemorrhage, malperfusion and systemic stress. Because current stage-stratified evidence is predominantly cross-sectional, differences in T-cell composition, transcriptional state and clonality across disease stages should not be interpreted as a proven longitudinal immunological trajectory. The subacute phase (15–90 days), after anatomical and haemodynamic stabilisation, may be particularly informative for testing persistent or injury-amplifying T-cell responses, whereas chronic AD (>90 days) may more closely reflect immune persistence, vascular remodelling and resolution. The subacute phase should therefore be regarded as an investigational window for mechanistic testing rather than an established therapeutic window [15,36–39].

#### Experimental criteria for establishing causality

Testing a post-onset injury-amplifying function requires a design distinct from studies of disease initiation. Established medial injury or dissection should first be documented by serial imaging, predefined histopathological criteria or other validated stage markers. T-cell-restricted depletion, inducible genetic perturbation or pathway inhibition should then be initiated after lesion establishment. Outcomes should assess progression—such as false-lumen expansion, medial destruction, aortic dilatation, propagation, rupture or adverse remodelling—rather than disease incidence alone. Rescue or reconstitution experiments would substantially strengthen attribution of the observed effect to a defined T-cell population. Accordingly, post-lesion causal attribution requires temporally delayed, T-cell-restricted intervention linked to disease-progression endpoints; prophylactic or global pathway inhibition alone is insufficient. In human AD, direct functional evidence from aortic-wall T cells remains difficult to obtain because tissue is generally available only after lesion formation and viable specimens are limited. This limitation should not preclude causal assessment. Where direct testing is not feasible, causal inference should rely on convergent evidence from spatially resolved human tissue studies, feasible *ex vivo* functional assays, mechanistically concordant disease-relevant models, and post-lesion T-cell-restricted perturbation with rescue or target-engagement evidence.

### T-cell alterations and systemic immune activation: clinical and translational implications

Systemic inflammatory activation in severe AD cannot be attributed specifically to T cells because malperfusion, hypotension, haemorrhage, ischaemia-reperfusion injury and surgery are major competing drivers. Adaptive-innate immune interactions may contribute to inflammatory persistence, but their independent effect on distal organ injury has not been established. The proposed links between local aortic inflammation, systemic immune activation, and distal-organ injury are summarised in Figure 5; the independent contribution of T cells remains unresolved [1,2,4,36–39].

**Figure 5.**
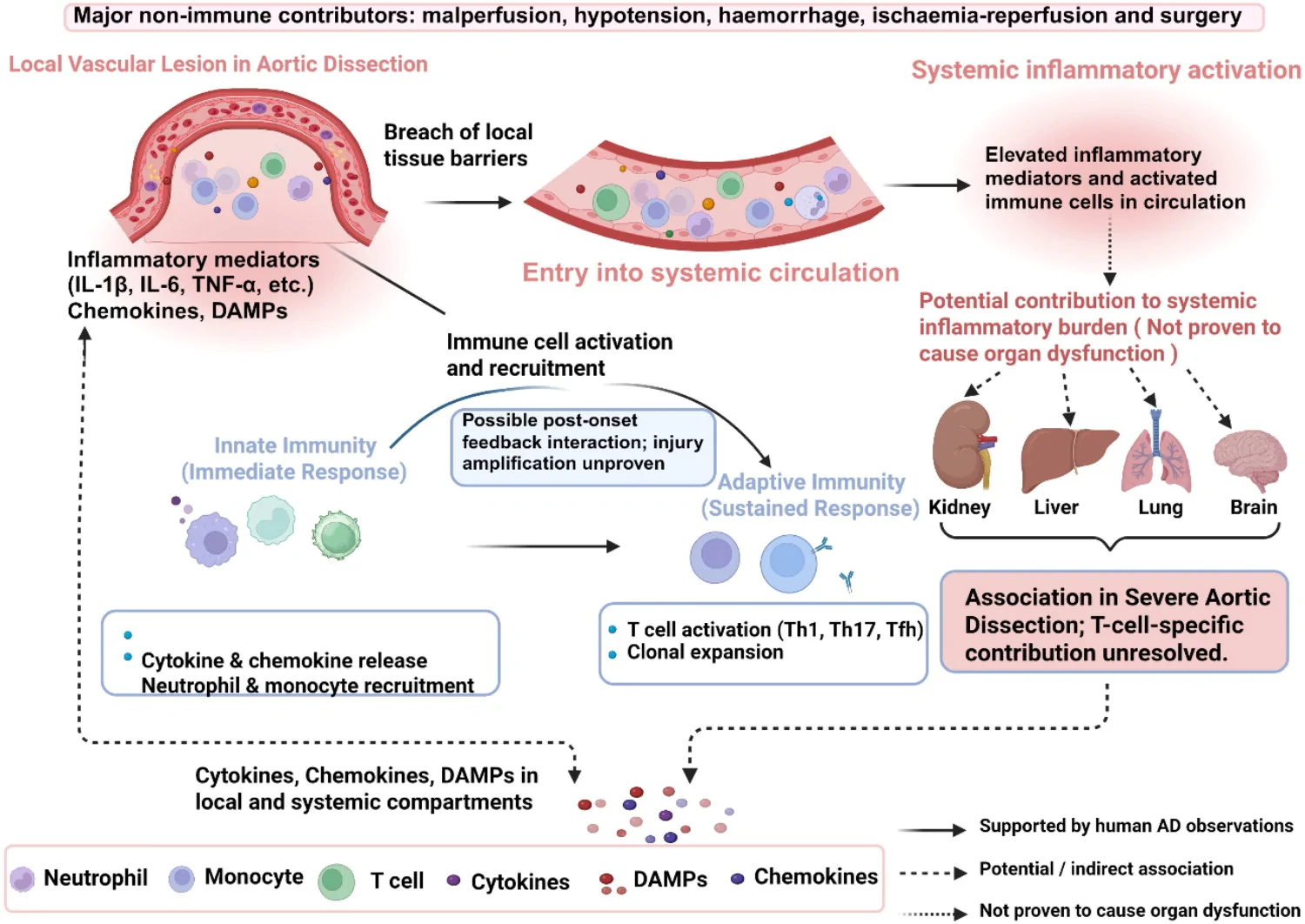
Potential links between local aortic inflammation, systemic immune activation, and distal-organ injury in severe aortic dissection. Dashed arrows indicate proposed or unvalidated relationships.

#### T-cell signatures as candidate biomarkers for disease progression and adverse aortic remodelling

At present, the most realistic clinical application of T-cell profiling in AD is risk stratification rather than therapeutic selection. Candidate signals include stage-associated changes in T-cell composition, cytotoxicity-associated CD8^+^ T-cell states, circulating helper/regulatory T-cell imbalance and, where TCR data are available, repertoire-based measures [15,16,28,29]. Circulating Th17-related measures and T-cell-associated inflammatory signals have also been associated with short- and intermediate-term clinical outcomes in AD patients [29,73,74]. However, none of the proposed T-cell signatures has undergone multicentre external validation or demonstrated reproducible incremental prognostic value beyond established clinical, imaging and routinely available inflammatory variables. Prospective studies should therefore use predefined sampling windows and serial blood collection, with paired aortic tissue where available, and relate immune measurements to clinically relevant outcomes such as false-lumen expansion, adverse aortic remodelling [75], reintervention and survival.

#### Implications for current and future therapeutic strategies

Current management of early acute AD remains centred on anatomical classification, complications and haemodynamic stabilisation, and immunomodulation has no established clinical role. Early acute and perioperative use is therefore unsupported, whereas the subacute phase may represent an investigational window after stabilisation. Translation will require post-lesion, T-cell-restricted validation, target engagement and acceptable stage-specific safety. Treg-directed strategies remain speculative because AD-specific efficacy and perioperative safety are unproven [76–79], while fludarabine has shown experimental anti-inflammatory effects but is not T-cell specific and lacks AD-specific clinical validation [80]. Among current candidates, the IL-6–STAT3–IL-17 axis has the strongest preclinical rationale for next-step post-lesion validation, as summarised in Table 3.

**Table 3.** Translational decision matrix for candidate T-cell-associated pathways in aortic dissection.

Pathway/candidate approach	AD-specific evidence	Major translational limitation	Temporal consideration	Priority for next-step validation
IL-6/IL-6R blockade	Experimental pathway-level evidence supports IL-6 signalling involvement, but available interventions do not establish post-lesion T-cell-specific efficacy [51,52].	Not T-cell specific; perioperative host-defence and vascular-repair effects remain uncertain.	Early acute/pre-stabilisation: not supported. Perioperative: not supported because AD-specific safety and effects on vascular repair remain uncertain. Subacute after stabilisation: investigational if persistent pathway activation and target engagement are demonstrated.	Priority for post-lesion mechanistic validation within the IL-6–STAT3–IL-17 axis.
IL-17A blockade	Experimental AD evidence supports pathway involvement, but the relevant cellular source and post-lesion effect remain unresolved [51,53].	Multiple cellular sources; antimicrobial host-defence effects and AD-specific perioperative safety remain uncertain.	Early acute/pre-stabilisation: not supported. Perioperative: not supported because AD-specific efficacy and safety remain unestablished. Subacute after stabilisation: investigational only, pending clarification of the relevant cellular source and post-lesion effects.	Priority for T-cell-source attribution and post-lesion functional validation.
JAK-STAT3 inhibition	Limited experimental AD evidence; available studies are neither T-cell restricted nor post-lesion [51].	Broad immune and vascular effects; systemic and perioperative safety in AD remains undefined.	Early acute/pre-stabilisation: not supported. Perioperative: not supported because systemic safety and effects on vascular repair remain uncertain. Subacute after stabilisation: investigational, pending post-lesion, cell-restricted validation.	Priority for cell-restricted validation and confirmation across independent AD models.
Low-dose IL-2/Treg expansion	No AD-specific efficacy data.	Uncertain selectivity, dose dependence, and perioperative safety.	Early acute/pre-stabilisation: not supported. Perioperative: not supported because AD-specific efficacy and safety are unknown. Subacute after stabilisation: investigational if persistent Treg dysfunction and selective target engagement are demonstrated.	Requires AD-specific proof-of-concept before prioritisation.
Adoptive Treg augmentation	No AD-specific efficacy data.	Manufacturing time, uncertain aortic homing, phenotypic stability, and perioperative immune safety.	Early acute/pre-stabilisation: impractical because of cell-manufacturing requirements. Perioperative: unsupported, with efficacy and immune safety unestablished. Subacute after stabilisation: investigational only, pending AD-specific efficacy, feasibility, and safety data.	Low immediate translational priority because of absent AD-specific efficacy data and major feasibility constraints.

*Note:* Evidence from non-AD settings is included only to provide mechanistic or safety context and should not be interpreted as direct evidence for therapeutic efficacy or safety in AD. These strategies are presented as mechanistic or preclinical hypotheses rather than treatment recommendations. The IL-6–STAT3–IL-17 axis currently provides the strongest rationale for further preclinical validation, but no candidate pathway is ready for clinical translation. The subacute phase is considered an investigational rather than an established therapeutic window. Detailed mechanistic and safety considerations are provided in Supplementary Table S3.

#### Challenges and safety considerations

The translational feasibility of immunomodulation is stage dependent. Early acute and perioperative use is currently unsupported, and perioperative immunosuppression raises infection-related safety concerns in non-AD surgical populations [81,82]. After anatomical and haemodynamic stabilisation, the subacute phase may provide a setting for experimental evaluation of persistent immune activation; however, AD-specific effects on vascular repair, postoperative outcomes, and therapeutic efficacy remain undefined. This phase should therefore be regarded as an investigational rather than an established therapeutic window. In acute AD, the injured aortic wall requires rapid structural repair. Systemic IL-6R or IL-17A blockade may compromise host defence and increase susceptibility to infection, particularly in the perioperative setting [81–84]. Any immunomodulatory strategy in acute AD must be evaluated in the context of emergency surgery, prosthetic graft implantation, bleeding risk, transfusion, mechanical ventilation, and postoperative infection susceptibility [39,82]. Furthermore, because inflammatory signalling also participates in tissue repair, excessive immunosuppression could theoretically affect vascular healing. This concern has not been evaluated directly in AD-specific studies. Consequently, preclinical studies should assess infection risk, perioperative safety, effects on vascular repair, target specificity and systemic immune suppression.

## Conclusion and outlook

Current evidence supports the presence of altered T-cell composition and transcriptional phenotypes in human AD after clinical onset, but it does not establish whether these responses initiate dissection, amplify injury after lesion formation, or represent bystander, reparative or resolution-associated immunity. In particular, cytotoxicity-associated gene expression, altered regulatory T-cell signatures and clonotype expansion should not be interpreted as evidence of vascular-cell killing, loss of suppressive function or aortic antigen specificity without direct functional validation. The next phase of the field should move beyond increasingly detailed immune-state catalogues toward causal attribution. Stage-defined human cohorts combining paired single-cell RNA/TCR profiling with spatial clonotype localisation can identify reproducible candidate populations. Causal inference should be strengthened through convergent human and experimental evidence, particularly when direct functional assessment of aortic-wall T cells is not feasible. Antigen specificity, where relevant, will require direct recognition assays rather than inference from TCR clonality alone.

This distinction between disease initiation and post-onset progression is critical, because prophylactic or global cytokine blockade cannot determine whether T cells actively propagate an established dissection. Among the candidate pathways, the IL-6–STAT3–IL-17 axis currently provides the strongest AD-specific rationale for next-step preclinical causal validation, although it is not ready for clinical translation.

Current evidence therefore does not support systemic T-cell-directed immunomodulation during the early acute and perioperative phases of AD. A more immediate translational question is whether reproducible circulating or tissue-associated T-cell signatures provide prognostic information beyond established clinical and imaging variables, particularly for adverse aortic remodelling and disease progression. Demonstrating causal vascular effects or independent prognostic value would provide the foundation for moving T-cell biology in AD beyond descriptive immune profiling toward clinically relevant stratification and, ultimately, targeted intervention. The field will move forward only if descriptive immune profiling is followed by causal validation and prospective clinical stratification, rather than by premature therapeutic extrapolation.

## Supplementary Material

Supplementary Material.docx

## Data Availability

This structured narrative review did not generate or analyse new datasets. All information discussed is derived from the cited literature.

## Patient and public involvement

No patients or members of the public were involved in the design, recruitment, conduct or dissemination of this study.
