# Supplementary material for: T-cell states in aortic dissection: from molecular immune profiling toward causal and translational validation
Source: Ann Med. 2026 Oct 29;58(1):2753327. doi: 10.1080/07853890.2026.2753327 (PMC13721806; doi:10.1080/07853890.2026.2753327)
Supplement: Supplementary file 1 [file IANN_A_2753327_SM0821.docx]

**Supplementary Table S1. Detailed search strategies used for the literature review.**

| **Database** | **Search strategy** | **Search period** | **Filters/limits** | **Records retrieved** | **Last search date** |
| --- | --- | --- | --- | --- | --- |
| PubMed | ("Aortic Dissection"[Mesh] OR "aortic dissection"[Title/Abstract] OR "aortic dissections"[Title/Abstract] OR "thoracic aortic dissection"[Title/Abstract] OR "acute aortic dissection"[Title/Abstract] OR "type A aortic dissection"[Title/Abstract] OR "type B aortic dissection"[Title/Abstract] OR "Stanford type A aortic dissection"[Title/Abstract] OR "Stanford type B aortic dissection"[Title/Abstract]) AND ("T-Lymphocytes"[Mesh] OR "T-Lymphocyte Subsets"[Mesh] OR "Receptors, Antigen, T-Cell"[Mesh] OR "T cell*"[Title/Abstract] OR "T lymphocyte*"[Title/Abstract] OR "CD4 T cell*"[Title/Abstract] OR "CD8 T cell*"[Title/Abstract] OR "helper T cell*"[Title/Abstract] OR "regulatory T cell*"[Title/Abstract] OR Treg*[Title/Abstract] OR Th1[Title/Abstract] OR Th17[Title/Abstract] OR "cytotoxic T cell*"[Title/Abstract] OR "T cell receptor*"[Title/Abstract] OR "TCR repertoire"[Title/Abstract]) | Database inception to 10 October 2026 | No language or publication-date restriction | 79 (Record counts represent database-specific yields before deduplication) | 10 October 2026 |
| Web of Science Core Collection | TS= ("aortic dissection"  OR "aortic dissections"  OR "thoracic aortic dissection"  OR "acute aortic dissection"  OR "type A aortic dissection"  OR "type B aortic dissection"  OR "Stanford type A aortic dissection"  OR "Stanford type B aortic dissection")  AND  TS= ("T cell*"  OR "T lymphocyte*"  OR "CD4 T cell*"  OR "CD8 T cell*"  OR "helper T cell*"  OR "regulatory T cell*"  OR Treg*  OR Th1  OR Th17  OR "cytotoxic T cell*"  OR "T cell receptor*"  OR "TCR repertoire") | Database inception to 10 October 2026 | No language or publication-date restriction | 70 (Record counts represent database-specific yields before deduplication) | 10 October 2026 |

**Note:** The PubMed search combined MeSH terms with Title/Abstract free-text terms, whereas the Web of Science Core Collection search used topic-based free-text terms adapted to the database syntax. Reference lists of eligible original studies and relevant reviews were manually screened for additional studies. Non-AD studies were used only to provide mechanistic context and were not assigned to the H1–H4 human AD evidence categories.

**Supplementary Table S2. Detailed mechanistic evidence supporting T-cell-associated findings in aortic dissection.**

| **T-cell finding** | **Experimental AD evidence** | **Mechanistic evidence from non-AD settings** | **Main limitation** | **Key references** |
| --- | --- | --- | --- | --- |
| Th17/IL-17-associated phenotype | Global IL-6/STAT3/IL-17A perturbation modifies selected AD-related outcomes, but available interventions were not T-cell restricted and were largely prophylactic or peri-induction. | Broader vascular studies support a role for Th17/IL-17 signalling in inflammatory vascular dysfunction, although its effects are context dependent and cannot be directly extrapolated to AD. | Existing AD studies do not establish a post-lesion, T-cell-specific IL-17A effect or direct Th17-mediated injury to aortic-wall cells. | AD evidence: [51-53]; non-AD mechanistic context: [42,44]. |
| CD8⁺ cytotoxicity-associated phenotype | No thoracic AD study has demonstrated CD8⁺ T-cell-specific depletion, rescue, reconstitution, or direct aortic target-cell validation. | Perforin–granzyme and Fas–FasL-mediated vascular-cell injury, together with CD8⁺ T-cell effector mechanisms, are derived mainly from atherosclerosis, AAA, and general cytotoxic T-cell biology. | Aortic antigen specificity, direct vascular-cell killing, and post-lesion CD8⁺ T-cell causality remain unproven. | [40-42,67] |
| Th1/IFN-γ-associated phenotype | No convincing T-cell-specific gain- or loss-of-function study has established a causal Th1/IFN-γ role in AD. | IFN-γ-associated changes in VSMC contractile, inflammatory, and matrix-related programmes are supported mainly by atherosclerosis and other non-AD vascular models. | The relevant cellular source, post-lesion timing, and direct effects of T-cell-derived IFN-γ on AD aortic-wall cells remain unresolved. | [57,58] |
| Treg-associated phenotype | No AD-specific study using Treg depletion or augmentation with functional rescue was identified. | Treg suppressive dysfunction, phenotypic instability, and plasticity are supported mainly by autoimmune and chronic inflammatory disease models. | AD-specific suppressive dysfunction, FOXP3 stability, lineage conversion, and causal effects on disease progression have not been established. | [68-72] |
| Antigen-specific immunity | No antigen-restricted AD model, cloned-TCR validation, or aortic target-cell recognition assay has established antigen-specific T-cell causality. | Experimental approaches for defining antigen-specific T-cell responses, including peptide–MHC recognition and cloned-TCR validation, are derived mainly from autoimmunity and myocarditis. | Clonotype expansion does not identify the relevant antigen or establish aortic autoreactivity; MHC-restricted recognition and target-cell effects remain unvalidated in AD. | [62-64] |
| Metabolic/epigenetic alterations | No T-cell-restricted metabolic or chromatin perturbation with rescue has been reported in AD models; available AD metabolic studies are predominantly tissue- or VSMC-focused. | T-cell metabolic reprogramming and epigenetic regulation are supported mainly by cancer, autoimmunity, and other inflammatory settings. | Aortic-wall T-cell metabolite concentrations, metabolic flux, chromatin state, and causal metabolic or epigenetic mechanisms remain uncharacterised. | AD metabolic evidence: [17-20]; non-AD T-cell mechanistic context: [21-27]. |

**Note:** Experimental AD evidence refers to mechanistic studies performed in disease-relevant AD models. Evidence from non-AD settings is included only to provide biological context and should not be interpreted as direct evidence for AD. Global or prophylactic pathway perturbation does not establish T-cell-specific or post-lesion causality.

**Supplementary Table S3. Detailed translational considerations for candidate immunomodulatory pathways relevant to aortic dissection.**

| **Pathway / candidate approach** | **Mechanistic rationale** | **T-cell specificity** | **Safety and repair concerns** | **Current translational status** | **Key references** |
| --- | --- | --- | --- | --- | --- |
| IL-6/IL-6R blockade | Inhibition of IL-6 signalling may reduce downstream STAT3 activation, Th17-favouring inflammatory programmes, macrophage recruitment, and inflammatory responses in vascular-wall cells. Experimental AD studies support pathway involvement, but available interventions do not establish post-lesion therapeutic efficacy. | Not T-cell specific. IL-6 signalling affects T cells, myeloid cells, endothelial cells, VSMCs, and systemic acute-phase responses. | Perioperative infection and wound-healing effects remain uncertain, and AD-specific safety data are unavailable. | AD-relevant preclinical rationale only. No post-lesion, T-cell-restricted efficacy or clinical evidence in AD. | AD mechanistic evidence: [49–52]; non-AD perioperative safety context: [81]. |
| IL-17A blockade | IL-17A inhibition may attenuate neutrophil recruitment, inflammatory cytokine amplification, matrix-degrading activity, and adverse extracellular-matrix remodelling. Experimental AD models support pathway-level involvement, but do not establish a post-lesion T-cell-specific effect. | Not strictly T-cell specific. IL-17A may derive from Th17 cells and other innate-like lymphocyte populations. | Potential effects on antimicrobial host defence remain relevant considerations; AD-specific perioperative safety data are unavailable. | Preclinical only. No AD-specific clinical evidence, and the relevant cellular source and post-lesion therapeutic effect remain unresolved. | AD mechanism: [51,53]; non-AD clinical safety context: [83,84]. |
| JAK-STAT3 inhibition | JAK-STAT3 inhibition may reduce cytokine-driven inflammatory transcription and Th17-associated differentiation or effector programmes. A STAT3 inhibitor reduced aortic dilatation and Th17 recruitment in an experimental model, but the intervention was neither delayed nor T-cell restricted. | Not T-cell specific. JAK-STAT3 signalling is shared by multiple immune and vascular-cell populations. | Broad non-T-cell-specific immune and vascular effects; systemic and perioperative safety in AD remains undefined. | AD-specific preclinical evidence is limited. No post-lesion, T-cell-restricted or clinical validation is available. | [48,51] |
| Low-dose IL-2 / Treg expansion | Preferential stimulation of high-affinity IL-2 receptor signalling may expand or support regulatory T cells and enhance suppressive immune activity. The rationale is derived primarily from non-AD immune-mediated diseases. | Treg-biased but not exclusively Treg specific. Depending on dose and biological context, IL-2 may also affect effector T cells and natural killer cells. | Dose sensitivity, unintended activation of non-Treg populations, systemic immune effects, and uncertain perioperative safety. | Clinically investigated outside AD but untested in AD. AD-specific efficacy, optimal dosing, target engagement, and perioperative safety remain undefined. | [76-78] |
| Adoptive Treg augmentation | Ex vivo-expanded autologous Tregs could theoretically suppress effector T-cell and myeloid inflammatory responses. However, no AD-specific efficacy data are available. | Relatively T-cell directed, but product purity, phenotypic stability, antigen specificity, and trafficking to the dissected aorta remain uncertain. | Manufacturing time, product variability, uncertain aortic homing, loss of suppressive phenotype, and uncertain perioperative immune safety. | Experimental and currently impractical for acute AD. AD-specific efficacy, durable suppressive function, tissue homing, and safety require demonstration before translational consideration. | [68,70,72,76,78] |

**Note:** Evidence from non-AD settings is included only to provide mechanistic or safety context and should not be interpreted as direct evidence for therapeutic efficacy or safety in AD. These strategies are presented as mechanistic or preclinical hypotheses rather than treatment recommendations. The IL-6–STAT3–IL-17 axis currently provides the strongest rationale for further preclinical validation, but no candidate pathway is ready for clinical translation. The subacute phase is considered an investigational rather than an established therapeutic window. Detailed mechanistic and safety considerations are provided in Supplementary Table S3.
